# Real-Time Imaging of the Epithelial-Mesenchymal Transition Using microRNA-200a Sequence-Based Molecular Beacon-Conjugated Magnetic Nanoparticles

**DOI:** 10.1371/journal.pone.0102164

**Published:** 2014-07-21

**Authors:** YoonSeok Choi, Hoe Suk Kim, Jisu Woo, Eun Hye Hwang, Kyoung-Won Cho, Soonhag Kim, Woo Kyung Moon

**Affiliations:** 1 Department of Biomedical Sciences, College of Medicine, Seoul National University, Jongro-gu, Seoul, Republic of Korea; 2 Department of Radiology, Seoul National University Hospital, Jongro-gu, Seoul, Republic of Korea; 3 The Institute of Radiation Medicine, Medical Research Center, Seoul National University, Jongro-gu, Seoul, Republic of Korea; 4 Department of Biomedical Sciences, College of Biomedical Sciences, CHA University, Gangnam-gu, Seoul, Republic of Korea; University of Colorado, Boulder, United States of America

## Abstract

The epithelial-mesenchymal transition (EMT) plays important roles in tumor progression to metastasis. Thus, the development of an imaging probe that can monitor transient periods of the EMT process in live cells is required for a better understanding of metastatic process. Inspired by the fact that the mRNA expression levels of zinc finger E-box-binding homeobox 1 (ZEB1) increase when cells adopt mesenchyme characteristics and that microRNA-200a (miR-200a) can bind to ZEB1 mRNA, we conjugated molecular beacon (MB) mimicking mature miR-200a to magnetic nanoparticles (miR-200a-MB-MNPs) and devised an imaging method to observe transitional changes in the cells during EMT. Transforming growth factor-β1 treated epithelial cells and breast cancer cell lines representing both epithelial and mesenchymal phenotypes were used for the validation of miR-200a-MB-MNPs as an EMT imaging probe. The real-time imaging of live cells acquired with the induction of EMT revealed an increase in fluorescence signals by miR-200a-MB-MNPs, cell morphology alterations, and the loss of cell-cell adhesion. Our results suggest that miR-200a-MB-MNPs can be used as an imaging probe for the real-time monitoring of the EMT process in live cells.

## Introduction

The epithelial-mesenchymal transition (EMT) is a process by which epithelial cells lose their abilities for cell-cell adhesion and gain invasive and motile properties. Although EMT is important for embryonic development and for the tissue repair process, it has been recognized as a potential mechanism of organ fibrosis and of tumor progression to metastasis [1], [2]. Previous reports have identified several EMT-related genes, such as zinc finger protein SNAI1 (SNAI1; Snail), zinc finger protein SNAI2 (SNAI2; Slug), zinc finger E-box-binding homeobox 1 (ZEB1; Zeb1), Twist-related protein 1 (TWIST1; Twist), and Cadherin-1 (CDH1; E-cadherin) [1]–[3]. Studies focused on ZEB1 and E-cadherin have revealed the interaction between these two genes as a key underlying mechanism of EMT [4]–[6]. In addition, members of the microRNA-200 family (miR-200a, b, c, -141, and -429) were reported as essential regulators in EMT because the repression of ZEB1 is mediated by their binding to the untranslated region (UTR) of ZEB1 mRNA [7]–[9]. Although several reports had assumed that the EMT might play a key role in tumor metastasis, the involvement of EMT in metastasis is still unclear due to the difficulties of cell behavior monitoring during EMT process [10]. Therefore, the development of EMT imaging methods is necessary, and, particularly, a method to trace the time points of changes in cellular characteristics from the epithelial to mesenchymal phenotype is expected to contribute the advancement of the knowledge on EMT process in tumor progression and metastasis.

A molecular beacon (MB) is a nucleic acid-based reporter probe that utilizes the principles of fluorescence resonance emission transfer (FRET) and releases fluorescence signals of a fluorophore from a quencher upon hybridization with a target oligonucleotide [11], [12]. The value of MBs ranges from measurements of DNA and RNA expression to molecular diagnosis and high-throughput genome screening [11]–[14]. In addition, MBs have been used as imaging probes for the direct observation of intracellular phenomena at the nucleic acid level because they can provide highly sensitive and dynamic images of nucleic acids in live cells [15]–[19]. Recently, MBs have also been used to demonstrate the movements of microRNAs or other classes of RNAs [20], [21]. Indeed, attempts at using MBs as a validated tool for monitoring the structures of nucleic acids or the biological functions of RNAs has proven their benefits in studying many molecular biological events [22]–[26]. To our knowledge, however, MBs or imaging probes for the EMT process in live cells has not been reported.

In this report, we employed an MB that mimics the mature miR-200a sequence to monitor the EMT process based on the facts that the expression level of ZEB1 mRNA notably increases during the EMT and that miR-200a can directly bind to the ZEB1 mRNA 3′-UTR. The miR-200a sequence-based MBs (miR-200a-MBs) were conjugated to polyethylene glycol (PEG) polymer-coated magnetic nanoparticles (miR-200a-MB-MNPs), and validations of miR-200a-MB-MNPs as EMT imaging probes were performed with multiple biochemical assays. Transforming growth factor-β1 (TGF-β1) treated mouse mammary epithelial cells (NMuMG) were used to acquire real-time imaging of the EMT process, and the results showed the feasibility of miR-200a-MB-MNPs as an imaging probe for monitoring the EMT process in live cells.

## Methods

### Cell culture

Human breast cancer cells (MCF-7 and MDA-MB-231; ATCC) were cultured in Dulbecco’s modified Eagle’s medium (DMEM; Invitrogen) containing 10% fetal bovine serum (FBS; Invitrogen). Normal mouse mammary (NMuMG; ATCC) and canine kidney (MDCK; KCLB) epithelial cells, well-known models for EMT studies, were also used [27]. MDCK cells were cultured in DMEM containing 10% FBS, and NMuMG cells were cultured in DMEM containing 10% FBS with 2 mM glutamine (Invitrogen) and 10 µg/ml insulin (Sigma). To induce EMT, NMuMG and MDCK cells were grown to 70–80% confluence, starved in DMEM containing 0.2% FBS for 12 hours, and treated with 10 ng/ml TGF-β1(R&D systems) up to 12 hours.

### Construction of miR-200a-MB-MNPs

**Figure 1:**
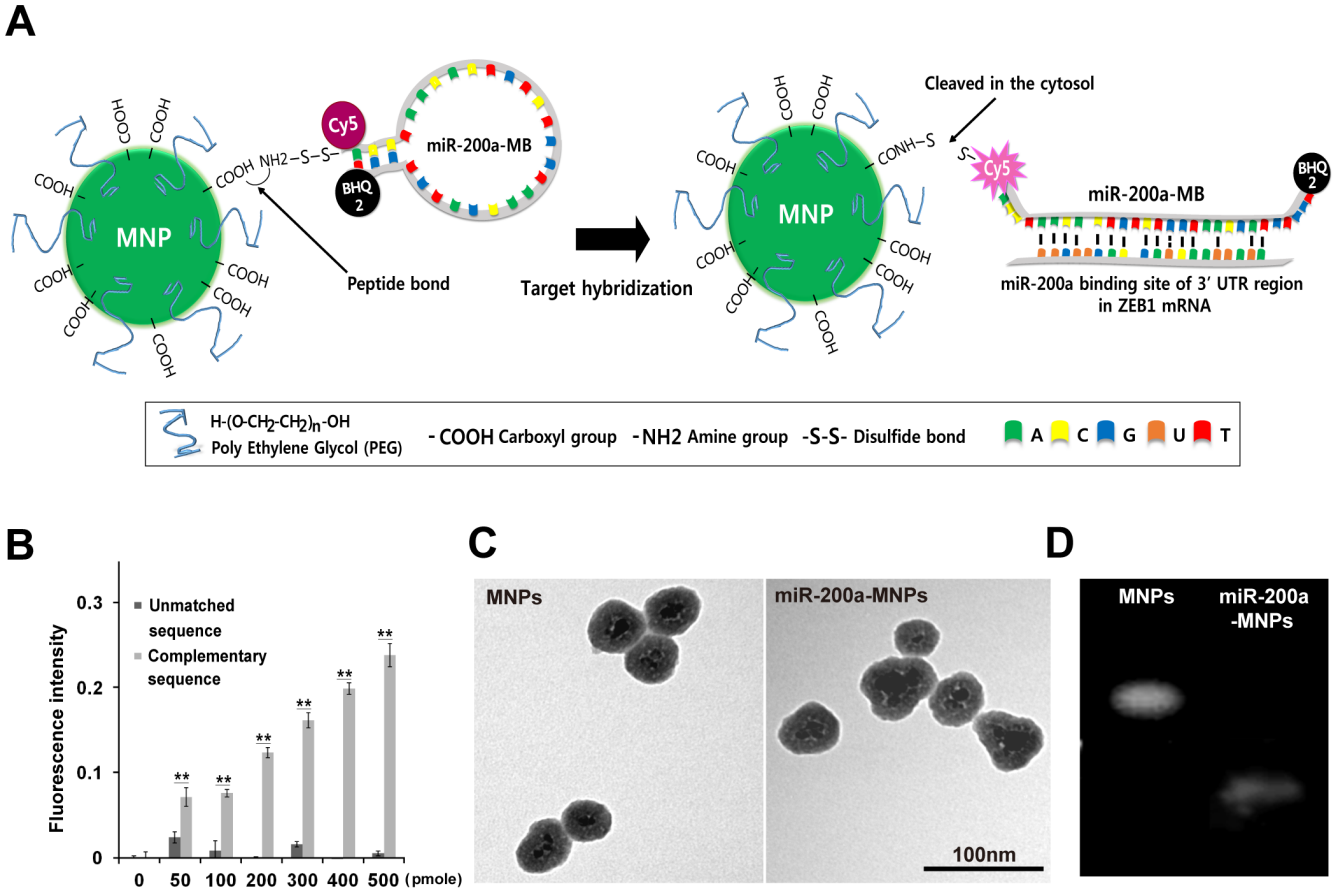
Characterization of miR-200a-MB-MNPs. (A) A schematic representation of the miR-200a-MB-MNP structure and EMT imaging probe activation. (B) Cy5 fluorescence from miR-200a-MBs. Different concentrations of a synthetic oligonucleotide target either complementary to or unmatched to miR-200a were added to the miR-200a-MBs. ** indicates p<0.01. (C) TEM images of MNPs and miR-200a-MB-MNPs. The diameters of the MNPs and miR-200a-MB-MNPs were approximately 50 nm. (D) An agarose gel electrophoresis image; MNPs and miR-200a-MB-MNPs were loaded onto 0.5% agarose gels.

Magnetic nanoparticles (MNPs) containing fluorescein isothiocyanate (FITC) and coated with a PEG polymer were purchased from Biterials. The surfaces of the MNPs were coated with carboxyl moieties (4.4×10^4^ per nanoparticle) and PEG (1×10^4^ per nanoparticle; 500 g/mol) to form PEG/COOH-MNPs. miR-200a-MBs were designed to have an amine group attached to the 5′ end of the oligonucleotide for the conjugation of miR-200a-MBs to MNPs. PEG polymers coated on MNPs were adapted to enhance the delivery of miR-200a-MBs. A disulfide-bond was included to allow the cleavage of miR-200a-MBs from MNPs within the cell cytoplasm. Cyanine 5 (Cy5) and black hole quencher 2 (BHQ2) were bound to the 5′ and 3′ ends of the oligonucleotide, respectively; this configuration emits a Cy5 signal only when the probe is bound to a complementary sequence (Fig. 1A).

The sequences of miR-200a-MB and the scrambled sequence molecular beacon (scrambled-MB) were as follows: miR-200a-MB, 5′-amine-S-S-A(Cy5)CCTAACACTGTCTGGTAACGATGTGGT-BHQ2-3′, and scrambled-MB, 5′-amine-S-S-G(Cy5)GACTCGTCTATGTTTCGGTAGTGTCC-BHQ2-3′ (Bioneer Inc.). The MNP carboxyl groups were covalently conjugated to the amine groups of miR-200a-MBs (20 µM) by incubating the molecules together for 1 hour at room temperature in the presence of N-ethyl-N′-dimethylaminopropyl carbodiimide (EDC; Sigma), which enhances the coupling efficiency between the amine and carboxyl groups. The miR-200a-MB-MNPs were centrifuged at 15,000 rpm for 10 minutes to remove the unconjugated free miR-200a-MBs and EDC, followed by several washes in Tris buffer (10 mM Tris-HCl, pH 7.4). After a brief sonication step, the final conjugated products were resuspended in phosphate-buffered saline (PBS, pH 7.4).

### *In vitro* fluorimetric assay

miR-200a-MBs were mixed with oligonucleotides containing sequences that are either complementary to miR-200a (ATTGTGTGACAGACCATTGCTACA) or an unmatched sequence (GAGCAGATACAAAGCCATC) (0–500 pmole) in 200 µl PBS. The miR-200a-MB and target oligonucleotide mixtures were incubated for 1 hour at 37°C and then transferred to opaque 96-well microplates. Their fluorescence intensities were measured using a Varioskan Flash microplate reader (Thermo Fisher Scientific).

### Gel electrophoresis

Gel electrophoresis was used to validate the conjugation of miR-200a-MBs to MNPs. A 50-µg sample of the miR-200a-MB-MNPs and unconjugated MNPs were subjected to electrophoresis through a 0.5% agarose gel using the Mupid-One (Advance) agarose gel electrophoresis system for 1 hour at 100 volts.

### Transmission electron microscope (TEM) imaging

TEM imaging of the miR-200a-MNPs and unconjugated MNPs was performed using a JEM 1010 microscope (JEOL Ltd.) at 80 kV. The miR-200a-MB-MNPs and unconjugated MNPs were fixed with 2% formaldehyde for 10 minutes, and the TEM digital images were recorded using a Gatan cooled charge-coupled device camera.

### Reverse transcriptase polymerase chain reaction (RT-PCR)

For RT-PCR analyses, RNA was isolated using the Trizol reagent (Invitrogen) and reverse transcribed using MMLV reverse transcriptase (New England Biolabs). The cDNA templates were amplified using GoTaq polymerase (Promega) for 25 cycles with following primer sets: E-cadherin – F, ATTCTGATTCTGCTGCTCTTG, and R, AGTAGTCATAGTCCTGGTCTT; ZEB1– F, TTCAAACCCATAGTGGTTGCT, and R, TGGGAGATACCAAACCAACTG; and GAPDH – F, CCTCTGGAAAGCTGTGGCGT, and R, TTGGAGGCCATGTAGGCCAT. The amplified products were subjected to electrophoresis through 1.5% agarose gels.

### Western blotting

Cells were lysed in RIPA buffer (Sigma), and the lysates were separated using SDS-polyacrylamide gel electrophoresis (SDS-PAGE) and transferred to nitrocellulose membranes. The membranes were blocked with 5% skim milk in Tris-buffered saline with Tween (TBST) and incubated with antibodies against E-cadherin (BD Biosciences), ZEB1 (Santa Cruz Biotechnology), or β-actin (Sigma) overnight at 4°C, followed by incubation with HRP-conjugated secondary antibodies (Santa Cruz Biotechnology). The blots were visualized using Enhanced Chemiluminescence Reagents (GE Healthcare Biosciences).

### Immunofluorescence staining

Cells were seeded onto sterile cover slips and placed in 24-well plates. miR-200a-MB-MNPs or scrambled molecular beacon-conjugated MNPs (scrambled-MB-MNPs) were added to the cells (20 µg/ml) and incubated for 30 minutes at 37°C. The cells were then fixed with a 4% paraformaldehyde solution (Sigma), and immunostaining for ZEB1, E-cadherin, Ago-2 or Rab7 was performed using anti-ZEB1 (Santa Cruz Biotechnology), anti-E-cadherin (BD Biosciences), anti-Ago-2 (Abcam) and anti-Rab7 (Santa Cruz Biotechnology) antibodies. The dye 4′,6-diamidino-2-phenylindole dihydrochloride (DAPI) was added for nuclear counterstaining (Invitrogen). All multicolor fluorescence images were obtained using confocal laser-scanning microscopy (LSM5 Meta; Carl Zeiss).

### Real-time PCR

miRNAs were isolated from cells using the mirVana miRNA isolation kit (Life Technologies), and primers for detecting miR-200a, miR-200b, and, miR-429 were purchased from Life Technologies. Reverse transcription was performed using the TaqMan microRNA reverse transcription kit (Life Technologies) according to the manufacturer’s instructions. 18s rRNA was used as an endogenous control. Real-time PCR of ZEB1 3′-UTR regions after the delivery of miR-200a-MNPs and scrambled-MNPs into EMT-induced NMuMG cells was also performed. The primers for the ZEB1 3′-UTR regions were F, GCTCTGACAGCCTTCCCG, and R, GTTTGGGAATTTTGACACAACA; the primers for GAPDH were the same primers described above. For assessment of effects of miR-200a-MBs-MNPs on the expression and stabilities of EMT-related transcription factors such as ZEB1, Snail and Slug, 1 µg/ml of actinomycin D (Sigma) was added into NMuMG cells after 2 hours of TGF-β1 treatment and RT-PCR analysis was performed with following primer sets: ZEB1 F, TTCAAACCCATAGTGGTTGCT, and R, TGGGAGATACCAAACCAACTG; Snail F, GAGGCGGTGGCAGACTAG and R, GACACATCGGTCAGACCAG; Slug F, CATGCCTGTCATACCACAAC, and R, GGTGTCAGATGGAGGAGGG were used. The ddCT method was used to calculate the miR-200 families, ZEB1, Snail and Slug levels.

### Bioluminescence imaging

For bioluminescence imaging, a Renilla luciferase fusion construct carrying the entire 3′-UTR of the ZEB1 mRNA (kindly provided by Dr. Gregory Goodall) was transfected into cells for 6 hours, and the cells were then cultured in growth medium for 24 hours. The transfected cells were screened for bioluminescence in complete media supplemented with 500 µg/ml coelenterazine (Promega) using the IVIS camera system (Xenogen). Imaging and signal quantification was performed using the acquisition and image-analysis software program Living Image (Xenogen).

### Real-time imaging

NMuMG cells were seeded onto the glass bottom dish and cultured to 50–60% confluence. When the cell numbers reached an appropriate number, miR-200a-MB-MNPs (20 µg/ml) were added to the cells and incubated for 30 minutes at 37°C in DMEM containing 0.2% FBS. TGF-β1 (10 ng/ml) was then added to the medium, and real-time imaging was acquired up to 20 minutes using confocal laser-scanning microscopy with the following parameters: FITC and Cy5 multi track; 20×/0.5 plan-neofluar; 512×512×1 stack size.

The analysis of the fluorescence signals generated by the miR-200a-MBs was performed with ImageJ software (National Instiute of Health). The color threshold range was selected to calculate the intensities of the Cy5 signal-bearing pixels, and every single image of the images serially acquired during the real-time imaging was analyzed by measurements of the pixel brightness.

### Statistical analysis

The data were analyzed for statistical significance using the two-tailed Student’s t-test for at least three independent experiments and are presented as the mean ± standard error of mean (S.E.M.). Differences were considered statistically significant at a value of p<0.05 or of p<0.01, indicated with asterisks (*, **).

## Results

### Characterization of molecular beacon-based imaging probes

As shown in Fig. 1A, miR-200a-MB contains the mature sequence of miR-200a and additional nucleotides to form a stem-loop structure, increasing the proximity of 5′-Cy5 and 3′-BHQ2. As the miR-200a-MB was designed to target ZEB1 mRNA, the Cy5 fluorophore is separated from BHQ2 and emits a fluorescence signal when miR-200a-MB hybridizes to a target sequence. As with the mouse ZEB1 mRNA sequence, the 3′-UTR regions of ZEB1 mRNAs across various species show the multiple miR-200a binding sites (Figure S1). In addition, the regions of miR-200a binding sites in the 3′-UTR regions of ZEB1 mRNAs indicate the strong possibility of miR-200a-MB binding to these target regions.

To evaluate the targeting ability of the miR-200a-MB, the Cy5 fluorescence intensity was measured after the addition of synthetic oligonucleotides containing either the miR-200a complementary sequence or an unmatched sequence. The Cy5 signals from the miR-200a-MBs increased following the addition of the miR-200a complementary sequence oligonucleotides in a dose-dependent manner, whereas the addition of unmatched sequence oligonucleotides did not increase the Cy5 fluorescence (Fig. 1B).

After the conjugation of miR-200a-MBs to MNPs, TEM images of the miR-200a-MB-MNPs were acquired. No disruption of PEG coated layers of miR-200a-MB-MNPs was observed compared to unconjugated MNPs (Fig. 1C). Electrophoresis was performed to verify that the miR-200a-MBs were conjugated to MNPs. Due to the extra negative charges of the conjugated oligonucleotides, the unconjugated MNPs and miR-200a-MB-MNPs migrated at different rates on the gel, as shown in Fig. 1D.

### Reciprocal expression of E-cadherin, ZEB1, and the miR-200 family during EMT

**Figure 2:**
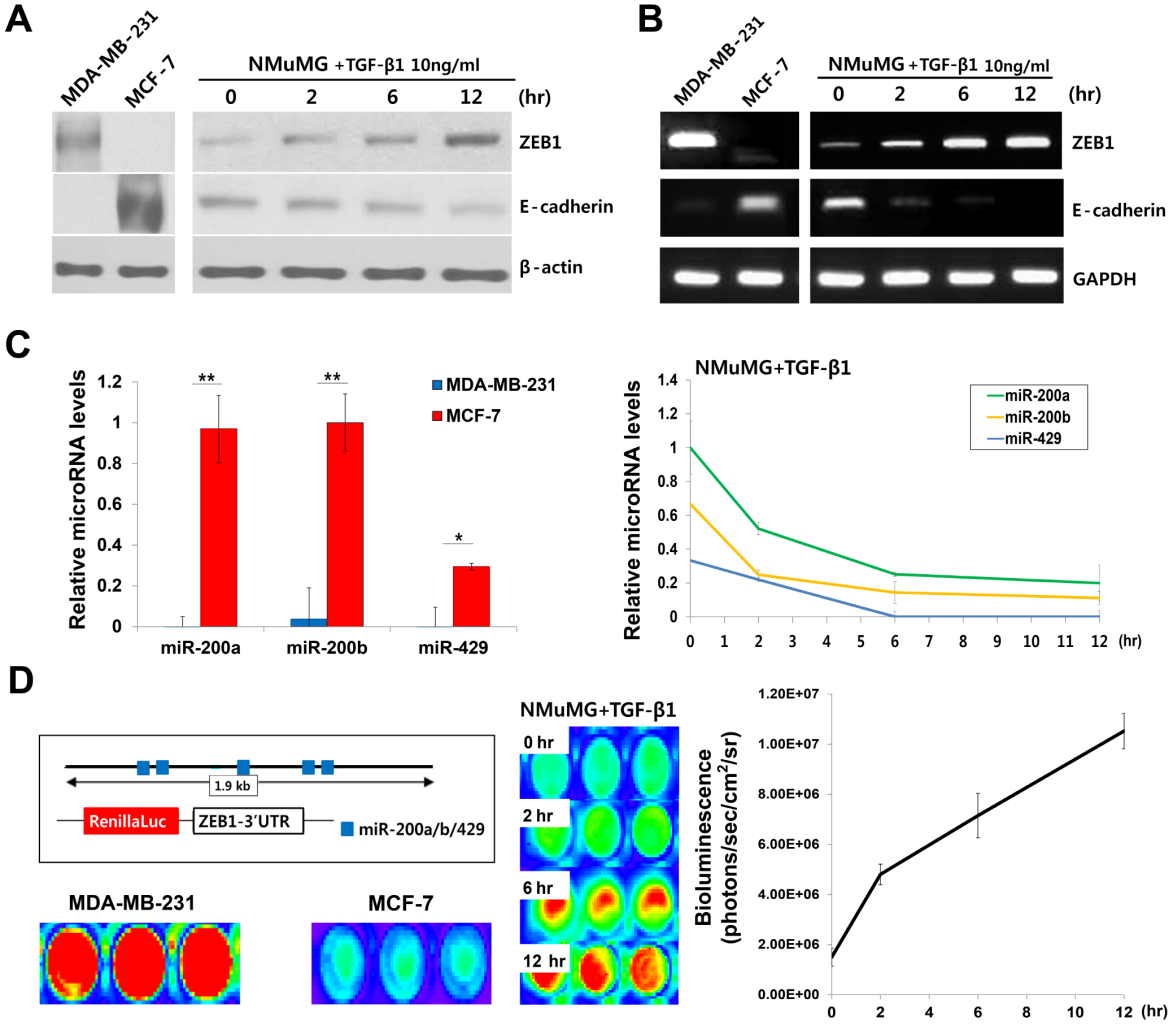
Reciprocal expression of E-cadherin, ZEB1, and the miR-200 family during EMT. (A, B) Western blot and RT-PCR analyses of ZEB1 and E-cadherin in MCF-7, MDA-MB-231, and TGF-β1 treated NMuMG cells. (C) Real-time PCR analysis of the miR-200 family. The levels of miR-200a, b, and 429 in MCF-7, MDA-MB-231, and TGF-β1 treated NMuMG cells were evaluated. *, ** indicates p<0.05 or p<0.01, respectively. (D) A schematic image of the Renilla luciferase reporter and the analysis of luminescence signals for evaluating the binding availability of miR-200a-MBs. After transfection of pRL-ZEB1 plasmid, the luminescence signals in MCF-7, MDA-MB-231, and TGF-β1 treated NMuMG cells were analyzed. Data are from three independent experiments.

Western blotting and real-time PCR were performed to investigate the reciprocal repression of E-cadherin, the miR-200 family, and ZEB1. Western blotting showed that E-cadherin was expressed in MCF-7 cells, whereas ZEB1 was expressed in MDA-MB-231. TGF-β1 treatment of NMuMG cells resulted in a time-dependent increase in ZEB1 expression and a concomitant decrease in E-cadherin expression (Fig. 2A).

We, next, evaluated the expression of ZEB1 and E-cadherin mRNA in MCF-7, MDA-MB-231, and TGF-β1 treated NMuMG cells. As shown in Fig. 2B, ZEB1 mRNA was only detected in the MDA-MB-231 cells, whereas E-cadherin mRNA was only detected in the MCF-7 cells. The NMuMG ZEB1 mRNA levels increased following TGF-β1 treatment and the induction of EMT, which was inversely correlated with E-cadherin mRNA expression. Expression levels of miR-200a, miR-200b, and miR-429 that are closely clustered in a chromosome and express as a single fragment precursor were also analyzed. miR-200 family members showed significantly lower expression levels in MDA-MB-231 compared to MCF-7 cells and were down-regulated in a time-dependent manner in TGF-β1 treated NMuMG cells (Fig. 2C).

To verify that the 3′-UTR regions of the ZEB1 mRNA are available for miR-200a binding, we performed luciferase imaging using the Renilla luciferase reporter. The bioluminescence of the transfected reporter was strongly repressed in the NMuMG and MCF-7 cells but was detectable in the TGF-β1 treated NMuMG and MDA-MB-231 cells (Fig. 2D). These results suggest that the ZEB1 mRNA 3′-UTR regions are available due to the loss of miR-200a expression and that the miR-200a-MBs could bind when the cells showed a mesenchymal phenotype.

### Validation of miR-200a-MB-MNP as an EMT-imaging probe

**Figure 3:**
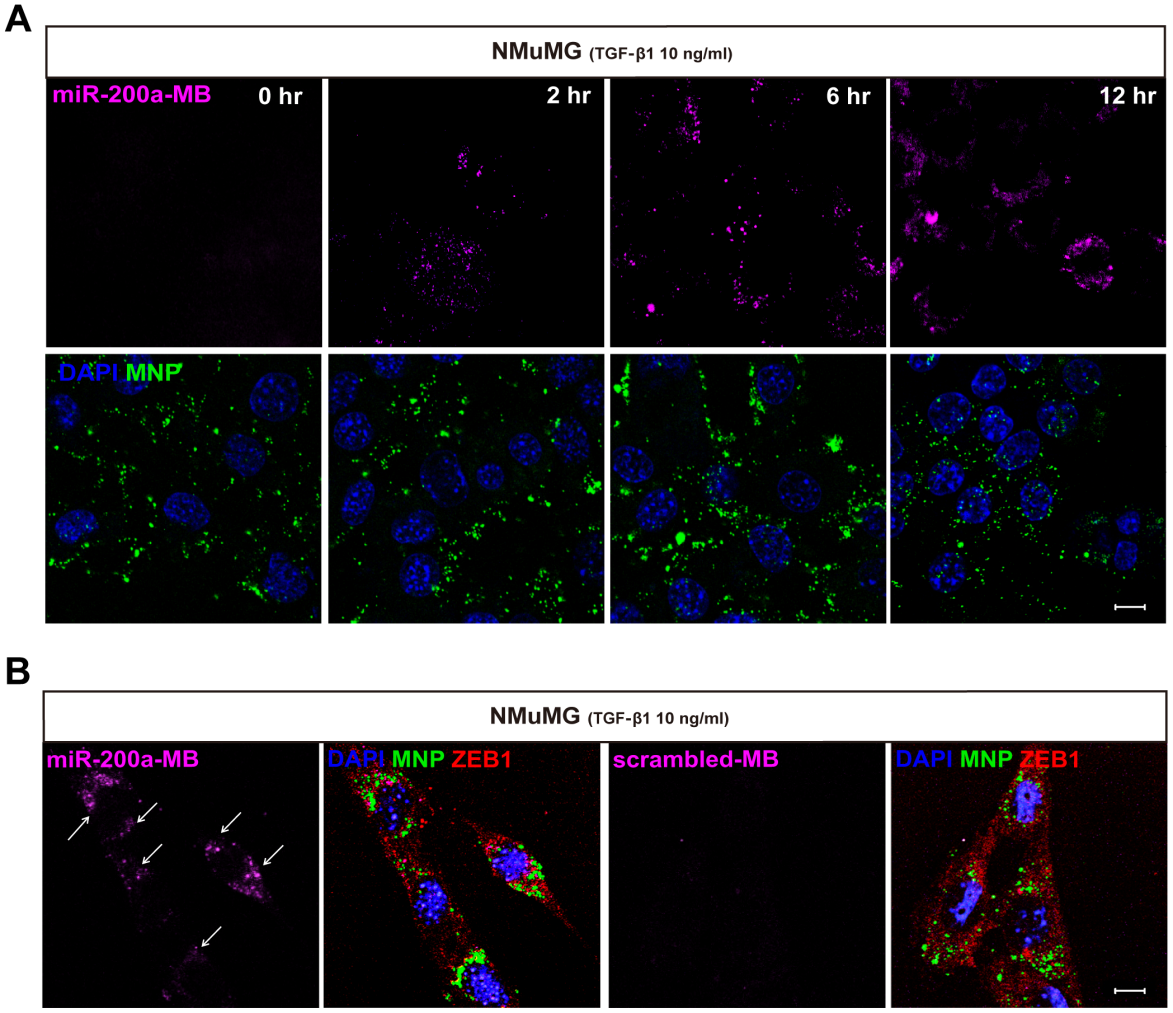
Validation of miR-200a-MB-MNPs as an EMT imaging probe. (A) miR-200a-MB-MNPs were introduced into NMuMG cells at 0, 2, 6, and 12 hours post-TGF-β1 treatment. Cy5 fluorescence (pink) was observed at 2 hours after TGF-β1 treatment, and the Cy5 signals increased in a time-dependent manner. (B) Cy5 signals were analyzed in either miR-200a-MB-MNPs or scrambled-MB-MNPs delivered NMuMG cells after 6 hours of TGF-β1 treatment. Immunostaining of ZEB1 was performed to confirm the mesenchymal transformation of NMuMG into mesenchymal phenotype. Scale bar, 10 µm.

We evaluated the possibility of miR-200a-MB-MNPs as an EMT-imaging probe by delivering them to TGF-β1 treated NMuMG cells for various incubation times (2, 6, or 12 hours); in all cases, miR-200a-MB-MNPs in the cytoplasm was confirmed by FITC fluorescence (Fig. 3A). Cy5 signals from the miR-200a-MBs were detected at 2 hours and increased in a time-dependent manner up to 12 hours post-treatment. To confirm whether the fluorescence signals were generated by miR-200a-MB binding and not by the generation of false positives, we analyzed the fluorescence of miR-200a-MB-MNPs and scrambled-MB-MNPs in NMuMG and MDCK cells following EMT induction with TGF-β1. Both the miR-200a-MB-MNPs and scrambled-MB-MNPs were internalized into the cytoplasm, and ZEB1 expression indicated that the NMuMG and MDCK cells were transformed to mesenchymal-type cells. Cy5 fluorescence was detected in the cells incubated with the miR-200a-MB-MNPs but not in those incubated with the scrambled-MB-MNPs (Fig. 3B, Figure S2A). Further validations of the miR-200a-MB-MNPs were analyzed using the representative epithelial and mesenchymal phenotype cells, MCF-7 and MDA-MB-231. The results showed that E-cadherin and ZEB1 were expressed in the MCF-7 and MDA-MB-231 cells, respectively. FITC fluorescence from the internalized MNPs was clearly detectable in the cytoplasm of both cell types, whereas Cy5 fluorescence, which was generated from the miR-200a-MBs, was only observable in the MDA-MB-231 cells (Figure S2B).

We next investigated whether miR-200a-MB-MNPs were taken up by the endosomal system and escaped from the endosomes in the cells. After 2 hours of TGF-β1 treatment, miR-200a-MB-MNPs were delivered to NMuMG cells and the localization of MNPs, MBs and Rab7, the late endosomal marker, were analyzed. After 5 minutes of delivery, light transmitted image and the fluorescence image of FITC signals showed the formation of endosomes and the encapsulation of MNPs into endosome (Figure S3). At 15 minutes after delivery of miR-200a-MB-MNPs co-localization of the MNPs and Rab7 was observed and the disruption of endosomal structures was found as the maturation of endosomes progressed (Figure S3).

### Hybridization of miR-200a-MBs and RNA-induced silencing complex and their influences to EMT program

**Figure 4:**
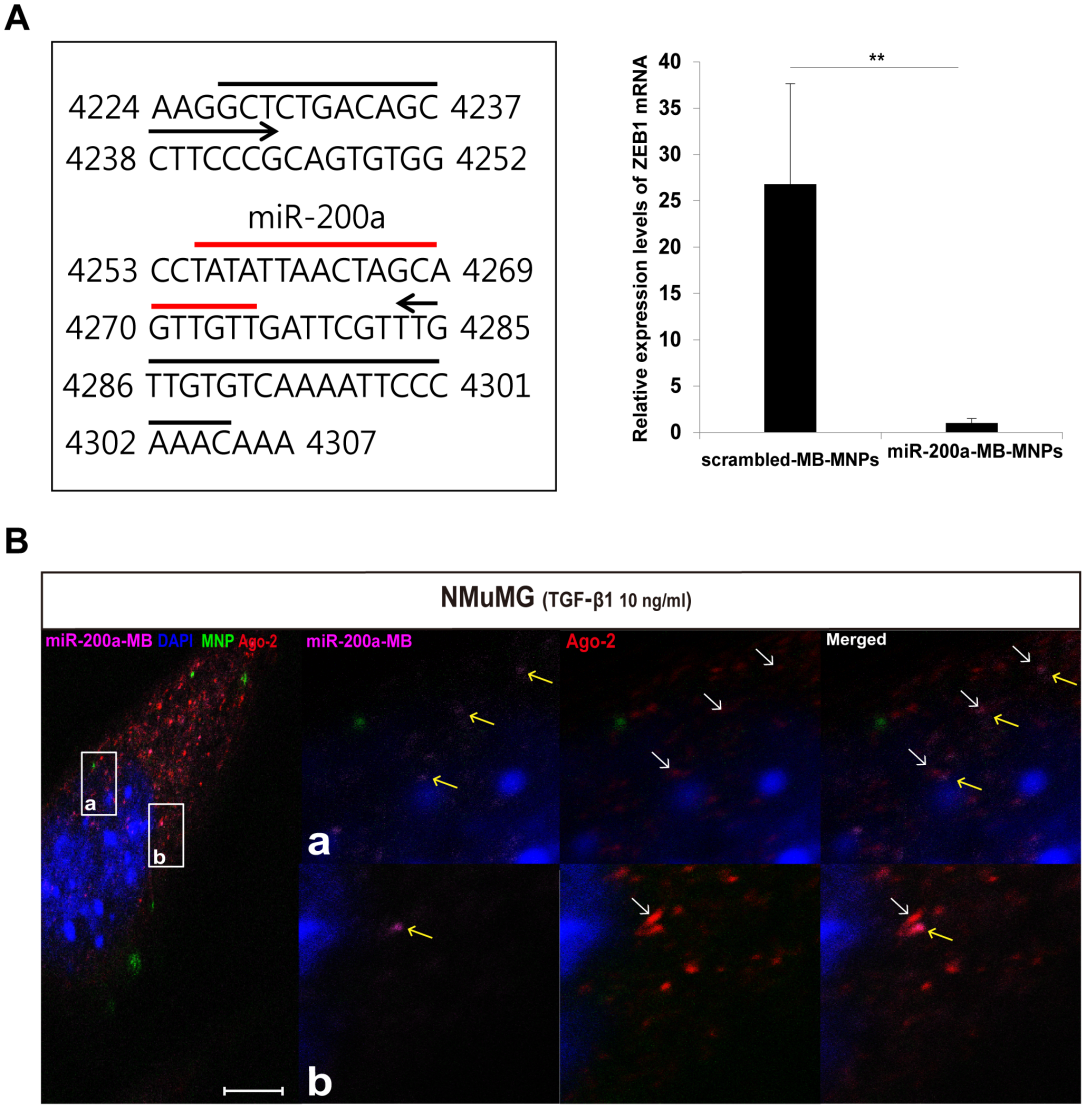
Analysis of the hybridization ability of miR-200a-MBs to ZEB1 mRNA using real-time PCR and immunostaining. (A) The illustration represents the binding sites of the primers used in real-time PCR. Real-time PCR analysis showed reduced expression levels of ZEB1 mRNA in miR-200a-MB-MNPs delivered NMuMG cells compared to the cells with scrambled-MB-MNPs. ** indicates p<0.01. (B) The localization of miR-200a-MBs and the RISC subunit Ago-2 were observed within the several regions of TGF-β1 treated cell. The Cy5 signals (yellow arrows) of miR-200a-MBs were in close proximity to the Alexa-546 signals of Ago-2 (white arrows). Small boxes were marked with a & b for the indication of the magnified regions. Scale bar, 10 µm.

The hybridization of miR-200a-MBs to the miR-200a binding sites of the ZEB1 mRNA was verified with real-time PCR and the localization of the MBs and RNA-induced silencing complex (RISC). We performed real-time PCR of the miR-200a binding sites to demonstrate that the miR-200a-MBs could be recruited after the delivery of both miR-200a-MBs and scrambled-MBs to EMT-induced NMuMG cells. Compared to the cells with scrambled-MB-MNPs, the NMuMG cells with miR-200a-MB-MNPs showed decreased amounts of ZEB1 mRNA, indicating the recruitment of miR-200a-MBs onto the 3′-UTR regions of the ZEB1 mRNA (Fig. 4A). In addition, we investigated the influences of the miR-200a-MBs on expression levels of EMT-related transcription factors such as ZEB1, Snail and Slug as well as the stabilities of these mRNAs in the presence of actinomycin D during the EMT processes. The mRNA levels of Snail and Slug were found to be increased by TGF-β1 in a time-dependent manner even after miR-200a-MB-MNPs delivery (Fig. S4). miR-200a-MB-MNPs-delivered?cells showed the decreased levels of ZEB1 mRNA at 30 minutes and 2 hours due to the binding of miR-200a-MB onto 3′-UTR regions of the ZEB1 mRNAs; however, ZEB1 mRNA levels showed an increment by TGF-β1. In the presence of actinomycin D the mRNA levels of ZEB1, Snail and Slug did not change significantly even though miR-200a-MB-MNPs were delivered into the cells (Fig. S4). The localization of miR-200a-MBs and Ago-2 was also analyzed to determine whether miR-200a-MBs can bind to RISC. Adjacent localizations of miR-200a-MBs and Ago-2 were detected in several areas of the cells and merged signals of the fluorescence of miR-200a-MBs and Ago-2 were also observed in the cytoplasm of cells (Fig. 4B). These results demonstrate that the miR-200a-MBs hybridize to ZEB1 mRNA without any influences on other EMT-related transcription factors.

### Real-time imaging of EMT with miR-200a-MB-MNPs

**Figure 5:**
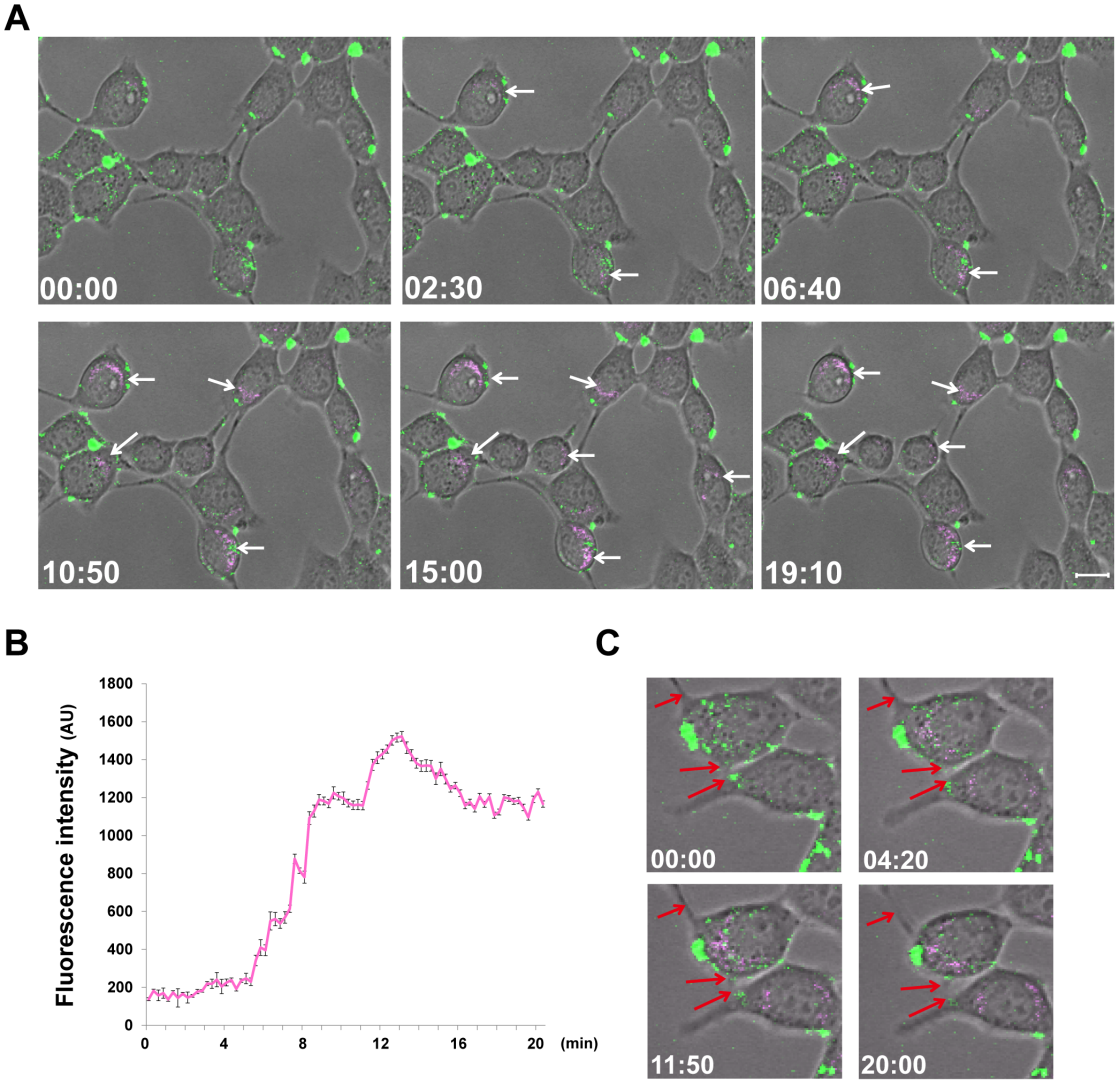
Real-time imaging of the 7EMT process in live cells. (A) After the delivery of miR-200a-MB-MNPs (20 µg/ml) into NMuMG cells, real-time imaging was performed following TGF-β1 (10 ng/ml) treatment. From 2 minute 30 seconds after EMT induction, NMuMG cells containing MNPs (green) in their cytoplasm showed Cy5 fluorescence (white arrows) generated by the miR-200a-MBs. (B) The Cy5 fluorescence intensity was quantified with Image J, showing increased signals generated by miR-200a-MBs. (C) During the acquisition period of real-time imaging, the NMuMG cells exhibited morphological changes and the loss of cell-cell adhesions (red arrows) after TGF-β1 treatment. Scale bar, 10 µm.

For the real-time imaging of EMT in live cells, miR-200a-MB-MNPs were introduced into NMuMG; after confirmation of miR-200a-MB-MNP internalization by the FITC fluorescence signals from the MNPs, TGF-β1 was used to induce EMT. At 2 minutes after EMT induction, NMuMG cells containing MNPs in their cytoplasm showed Cy5 fluorescence generated by the miR-200a-MBs, though the fluorescence intensity was faint. The quantification of the Cy5 signals showed that the signals across the entire imaging field gradually increased up to 20 minutes as EMT progressed (Fig. 5A, B, Movie S1).

We also observed NMuMG morphological changes in several areas; the loss of cell-cell adhesion was observed after EMT induction, and the degree of detachment increased in a time-dependent manner. Furthermore, the miR-200a-MB-MNP-delivered cells that showed altered morphology and the loss of cell-cell adhesion showed increases in Cy5 signals (Fig. 5C). This result indicated that the Cy5 signals generated by miR-200a-MB reflected the degree of the EMT process and implied that miR-200a-MB-MNPs can be used for the real-time monitoring of the EMT process in live cells.

## Discussion

The EMT process comprises a complex series of cell-biological events that culminate in changes in gene expression and cell morphology [28] and the duration of this process is presumed to short [29], [30]. Therefore, the development of an imaging tool for the real-time monitoring of the EMT process will be indispensable in understanding the dynamics of EMT regulation in live cells and for developing treatment strategies of EMT-related diseases such as organ fibrosis and tumor metastasis.

For imaging EMT process in live cells, the use of reporter genes instead of MBs could be considered. By fusing the EMT marker-gene promoter to reporter genes, the EMT process may be monitored with optical imaging systems [31], [32]. However, although the successful induction of these reporters in cells has been achieved, these techniques are affected by many variables and have limits in their ability to monitor the rapid and dynamic EMT process. Concerning the limits of the reporter gene-based imaging methods, MBs represent a potential imaging probe candidate for the direct monitoring of transient events in the EMT process because of their rapidness in signal emission to various stimuli from the cell environment. Although we only used TGF-β1 to induce EMT, our real-time imaging results showed that an MB-based imaging probe can provide a reliable method for monitoring the transient events and immediate response of cells during EMT. In particular, MBs that contain miR-200a sequence can serve as an excellent imaging probe because the levels of ZEB1 mRNA expression are drastically up-regulated during EMT, offering many sites for miR-200a-based MB binding. Additionally, as many previous studies have reported concrete evidence of miR-200a and ZEB1 mRNA interactions [7]–[9], 8- to 14-mers of matching sequence between the miR-200a-MB and ZEB1 mRNA appears to be sufficient to evoke fluorescence signals from miR-200a-MBs.

Following the rapid developments in nanotechnology, MNPs have been used to efficiently deliver drugs and genes into cells and tissues and to simultaneously image these processes *in vitro* and *in vivo* [33]–[36]. MNP are taken up by cells through endocytosis which is a complex process [37], [38] The endocytic uptake and endosomal escape mechanisms by which cells take MNP and release specific MNP from endosome would be of interest to the researchers in the field of nanomedicine, cancer diagnosis and treatment. miR-200a-MB-MNPs tend to rapidly accumulate inside endocytic organelles, escape from endosome stained with late endosomal marker, Rab7 and reach the cytosolic space efficiently. Several studies using MBs have suggested approaches to avoid false positives by the conjugation of MBs to MNPs [39]. Therefore, we utilized MNPs to avoid the detection of false-positive signals and also improved the delivery efficiency of miR-200a-MBs using PEG polymers on the MNP surface. We here suggest miR-200a-MB-MNPs go through endocytic route and many miR-200a-MBs survive in endosomes and cytosol after the release from the MNP. Furthermore, as MNPs can be tracked by their fluorescence, the conjugation of MNPs to miR-200a-MBs should enable future animal studies.

Even we designed the miR-200a sequence that can preferentially bind with ZEB1 mRNAs, miR-200a MBs have the potential to bind the other genes and can block the miR binding site on multiple mRNA related with the EMT process. In our study, however, the validation of miR-200a-MB-MNP as an EMT-imaging probe was performed by various biochemical assays. A comparative analysis by a fluorimetric assay and immunostaining with miR-200a-MBs and scrambled-MBs showed the targeting ability of miR-200a-MBs to ZEB1 mRNA. In addition, real-time PCR of the ZEB1 3′-UTR regions after miR-200a-MB-MNPs delivery and the localization of miR-200a-MB signals adjacent to Ago-2, an RISC complex subunit, verified the hybridization of miR-200a-MBs and the target mRNA. miR-200a-MB-MNPs did not alter the increase of EMT-related transcription factors, Snail and Slug except for ZEB1, and did not affect the stability of these mRNAs in miR-200a-MB-MNP-delivered cells during EMT-induction. As the miR-200a sequence is evolutionarily well conserved and the 3′-UTR of ZEB1 mRNA is also well conserved, it appears that miR-200a-MB-MNPs can be universally used to investigate the EMT process across species.

Although our study focused on preferential ZEB1 targeting by miR-200a, MBs that are conjugated to MNPs can be designed for other EMT-associated genes, such as SNAI1, SNAI2, and TWIST1. Other members of the microRNA-200 family or microRNAs that regulate the EMT process can also be designed as potential candidates for EMT imaging. Furthermore, though we utilized DNA-based MBs as many papers have shown the feasibility of real-time imaging with the hybridization of DNA-RNA and for their ease of handling due to their better stability versus RNA-based MBs [15], [40], modifications for 2′-O-methylribonucleotide-based backbones or to include locked nucleotides for the significant improvement in stability will efficiently enhance the targeting ability of MBs [41]–[43]. Additionally, the MNPs that we utilized as a protective carrier to protect against nucleases can further be modified to enhance the MB stability and moreover to possess and active targeting ability for the cell of interest [44]–[46]. These modifications will increase the usefulness of miR-200a-MB-MNPs as an imaging probe for the EMT process.

In conclusion, our study demonstrates the feasibility of miR-200a-MB-MNPs as an imaging tool for the real-time monitoring of the EMT process in live cells. Information provided by real-time imaging with miR-200a-MB-MNPs will advance the understanding of spatially regulated gene expression in EMT and the development of therapies for EMT-related diseases.

## Supporting Information

Figure S1 **Comparison of miR-200a binding sites in the ZEB1 mRNAs from multiple species.** Binding sites of miR-200a are evolutionarily well conserved among mouse, human, and canine.
(TIF)

Figure S2 **Validations of miR-200a-MB-MNPs as an EMT imaging probe in different cell lines.** (A) miR-200a-MB-MNPs and scrambled-MB-MNPs were introduced into TGF-β1 treated MDCK cells. Fluorescent signals from miR-200a-MBs (pink) were only detected in the miR-200a-MB-MNP-delivered MDCK cells. (B) E-cadherin (red) was expressed in MCF-7 cells, and ZEB1 (red) was expressed in MDA-MB-231 cells. Cy5 fluorescence signals (pink, white arrows) were only detected in the MDA-MB-231 cells and not in the MCF-7 cells. Scale bar, 10 µm.
(TIF)

Figure S3 **Endosomal escape of miR-200a-MB-MNPs.** miR-200a-MB-MNPs (20 µg/ml) were introduced into NMuMG cells after 2 hours of TGF-β1 treatment (10 ng/ml). Light transmitted image showed the endosome morphologies (white dashed circle) and encapsulated MNPs (green) were observed in the endosome after 5 minutes of delivery. Assembled late endosome marker, Rab7 (red, yellow arrows), around the MNPs indicated that MNPs were taken up by the endocytosis pathway in the cells. Disruption of the endosome morphologies (yellow dashed circle) and scattered miR-200a-MBs (pink) signals indicated that miR-200a-MBs were released from MNPs in the cytosol of NMuMG cells. Scale bar, 10 µm.
(TIF)

Figure S4 **Assessment of the effects of miR-200a-MB-MNPs on EMT-related transcription factors and the mRNA stabilities.** mRNA levels of EMT-related transcription factors were evaluated after the delivery of miR-200a-MB-MNPs into NMuMG cells after 2 hours of TGF-β1 treatment (10 ng/ml). EMT induced by TGF-β1 increased levels of ZEB1, Snail and Slug. ZEB1 mRNAs slightly decreased by the miR-200a-MB-MNPs after 30 minutes of delivery, however, showed increased expression levels after 2 hours of incubation. Stabilities of mRNAs of ZEB1, Snail and Slug did not differ significantly by the miR-200a-MB-MNPs. Act D = Actinomycin D.
(TIF)

Movie S1 **Real-time imaging of the EMT process.** Real-time imaging was acquired after the treatment of TGF-β1 (10 ng/ml) and miR-200a-MB-MNPs (20 µg/ml). An animation file in wmv format.
(WMV)
