# Supplementary figures and images for: Real-Time Imaging of the Epithelial-Mesenchymal Transition Using microRNA-200a Sequence-Based Molecular Beacon-Conjugated Magnetic Nanoparticles
Source: PLoS One. 2014 Jul 21;9(7):e102164. doi: 10.1371/journal.pone.0102164 (PMC4105468; doi:10.1371/journal.pone.0102164)

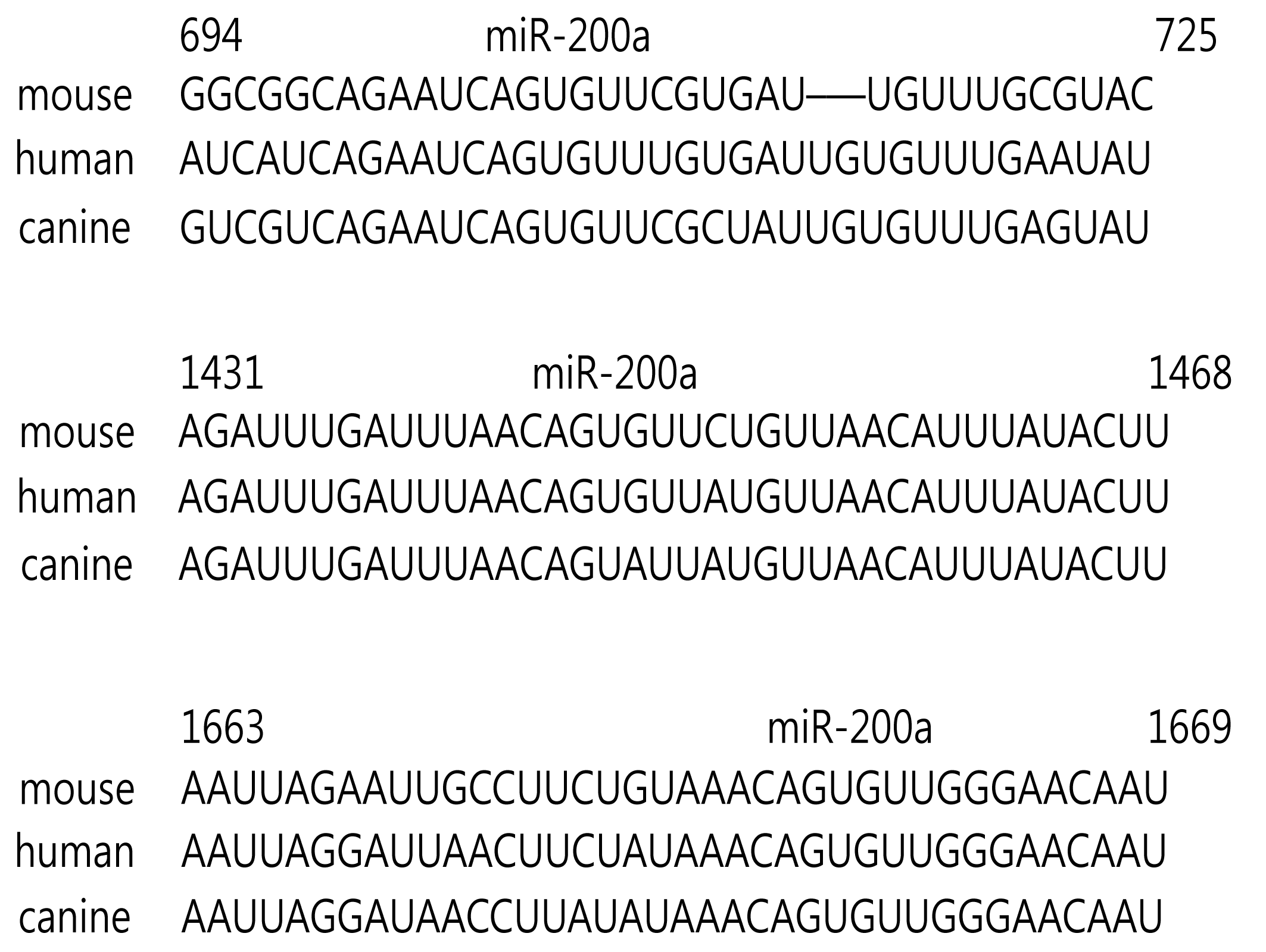

Supplement: Figure S1 — Comparison of miR-200a binding sites in the ZEB1 mRNAs from multiple species. Binding sites of miR-200a are evolutionarily well conserved among mouse, human, and canine. (TIF) [file pone.0102164.s001.tif]

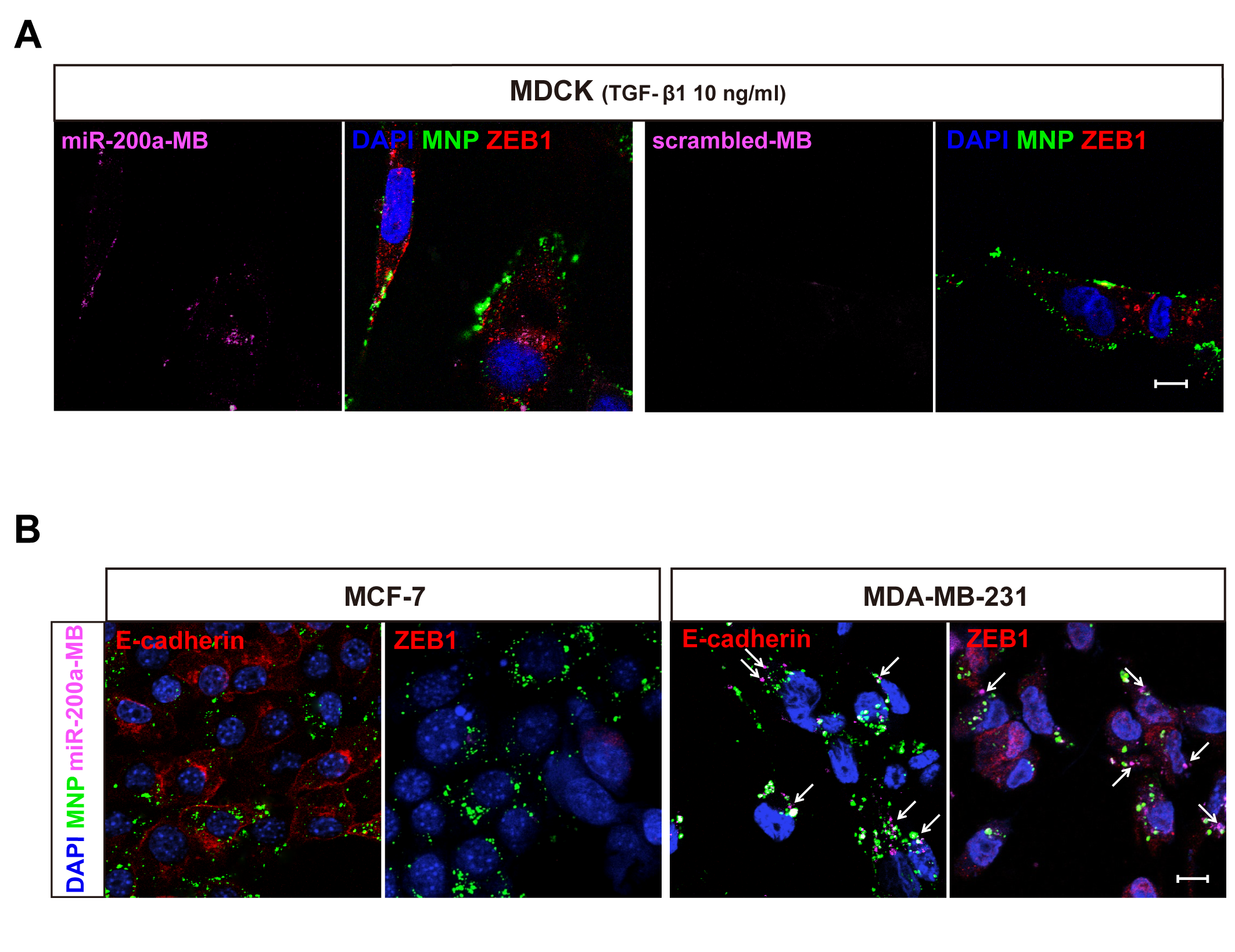

Supplement: Figure S2 — Validations of miR-200a-MB-MNPs as an EMT imaging probe in different cell lines. (A) miR-200a-MB-MNPs and scrambled-MB-MNPs were introduced into TGF-β1 treated MDCK cells. Fluorescent signals from miR-200a-MBs (pink) were only detected in the miR-200a-MB-MNP-delivered MDCK cells. (B) E-cadherin (red) was expressed in MCF-7 cells, and ZEB1 (red) was expressed in MDA-MB-231 cells. Cy5 fluorescence signals (pink, white arrows) were only detected in the MDA-MB-231 cells and not in the MCF-7 cells. Scale bar, 10 µm. (TIF) [file pone.0102164.s002.tif]

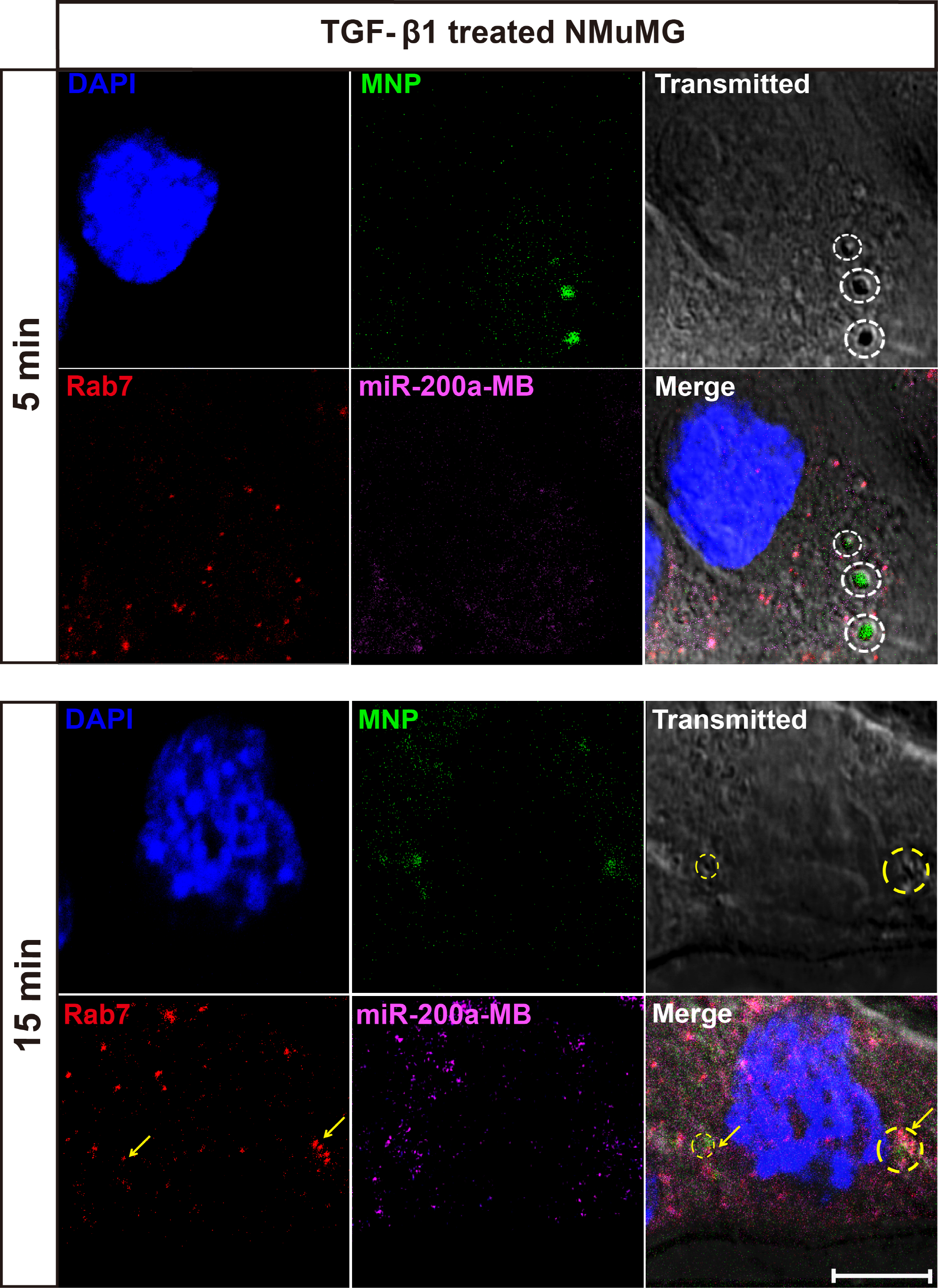

Supplement: Figure S3 — Endosomal escape of miR-200a-MB-MNPs. miR-200a-MB-MNPs (20 µg/ml) were introduced into NMuMG cells after 2 hours of TGF-β1 treatment (10 ng/ml). Light transmitted image showed the endosome morphologies (white dashed circle) and encapsulated MNPs (green) were observed in the endosome after 5 minutes of delivery. Assembled late endosome marker, Rab7 (red, yellow arrows), around the MNPs indicated that MNPs were taken up by the endocytosis pathway in the cells. Disruption of the endosome morphologies (yellow dashed circle) and scattered miR-200a-MBs (pink) signals indicated that miR-200a-MBs were released from MNPs in the cytosol of NMuMG cells. Scale bar, 10 µm. (TIF) [file pone.0102164.s003.tif]

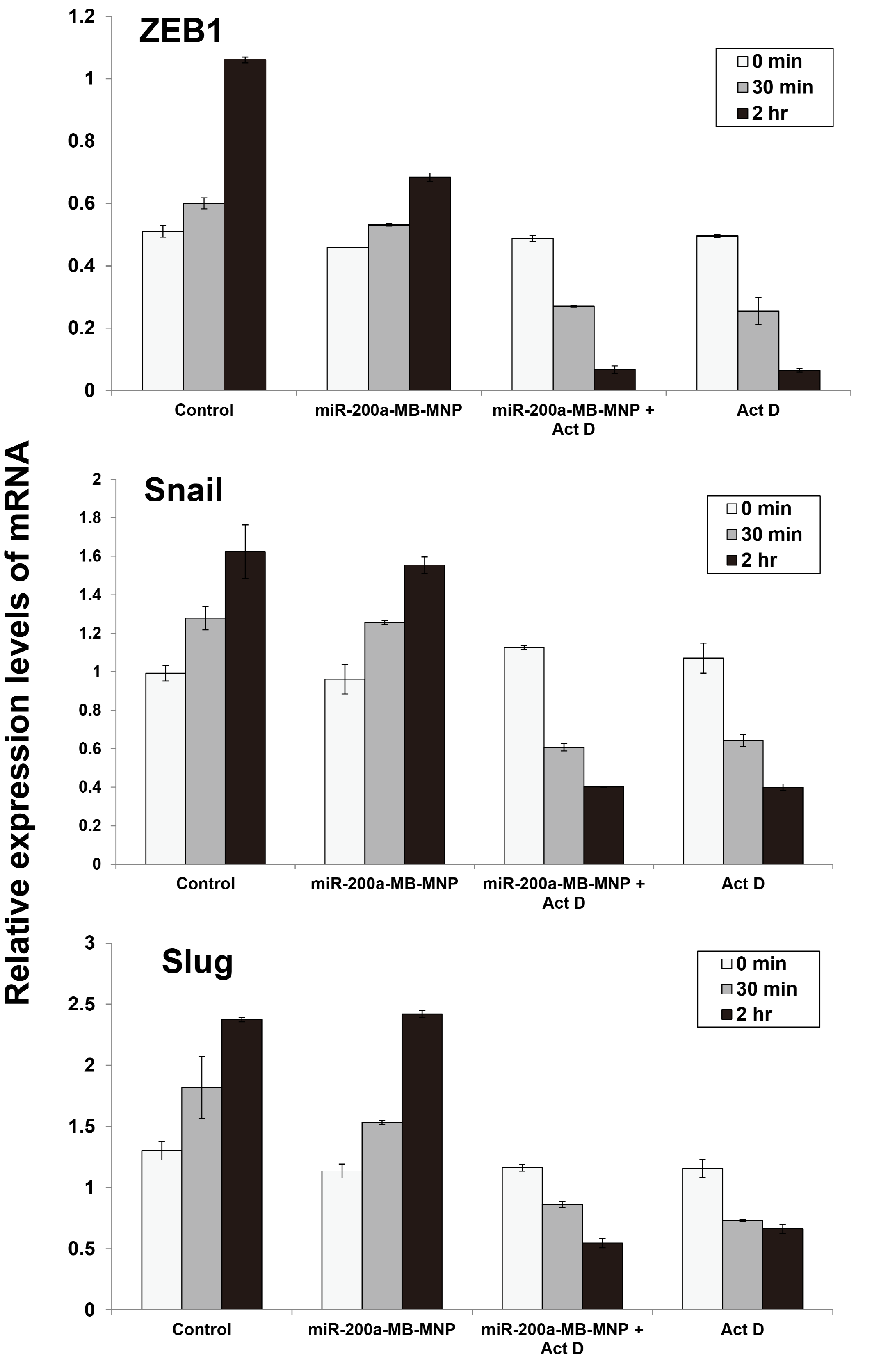

Supplement: Figure S4 — Assessment of the effects of miR-200a-MB-MNPs on EMT-related transcription factors and the mRNA stabilities. mRNA levels of EMT-related transcription factors were evaluated after the delivery of miR-200a-MB-MNPs into NMuMG cells after 2 hours of TGF-β1 treatment (10 ng/ml). EMT induced by TGF-β1 increased levels of ZEB1, Snail and Slug. ZEB1 mRNAs slightly decreased by the miR-200a-MB-MNPs after 30 minutes of delivery, however, showed increased expression levels after 2 hours of incubation. Stabilities of mRNAs of ZEB1, Snail and Slug did not differ significantly by the miR-200a-MB-MNPs. Act D = Actinomycin D. (TIF) [file pone.0102164.s004.tif]
